# Tree Shrew Genome-Wide CRISPR Screen Identifies RNF6 as a Proviral Host Factor for Zika Virus Replication in Brain Microvascular Endothelial Cells

**DOI:** 10.3390/v18030323

**Published:** 2026-03-05

**Authors:** Mengdi Qi, Xin Liu, Wenguang Wang, Meili Lu, Qingwei Zeng, Na Li, Yuanyuan Han, Shengtao Fan, Caixia Lu, Jiejie Dai

**Affiliations:** 1 Institute of Medical Biology, Chinese Academy of Medical Sciences & Peking Union Medical College, Kunming 650118, China; 2 School of Life Sciences, Yunnan University, Kunming 650500, China

**Keywords:** flavivirus, Zika virus, tree shrew, CRISPR screen, GeCKO library, host factor, RNF6

## Abstract

Zika virus (ZIKV), a unique flavivirus with neurotropic and teratogenic potential, can cross the blood–brain barrier and persist in human brain microvascular endothelial cells (BMECs); however, no approved vaccines or specific antivirals exist, and its barrier-crossing and neuroinvasive mechanisms remain elusive. Innovative strategies to identify additional host factors mediating ZIKV infection could yield key insights and help address these challenges. To uncover novel host factors, we established the first tree shrew (*Tupaia belangeri*) genome-wide CRISPR/Cas9 knockout (GeCKO) library and performed a screen in BMECs, identifying ring finger protein 6 (RNF6) as a novel proviral factor for ZIKV. ZIKV infection in BMECs was significantly reduced following RNF6 knockout or knockdown but enhanced upon RNF6 overexpression or rescue. Mechanistically, RNF6 interacts with the ZIKV NS5 protein and acts as a potential negative regulator of the type I interferon and MAPK signaling pathways. Evolutionary and structural analyses revealed that RNF6 is highly conserved between humans and tree shrews; molecular docking further identified shared NS5-binding residues (Gln-59, Arg-140), supporting the conserved proviral role of human RNF6 in ZIKV infection. Our findings highlight tree shrew GeCKO screening as an efficient approach for identifying novel host factors and establish RNF6 as a critical proviral factor for ZIKV replication in BMECs, providing new insights into ZIKV neurotropic pathogenesis and informing potential antiviral strategies.

## 1. Introduction

Emerging and re-emerging viruses, including severe acute respiratory syndrome coronavirus 2 (SARS-CoV-2), monkeypox virus (MPXV), and arthropod-borne viruses (arboviruses), continue to threaten global public health. Among these, Zika virus (ZIKV) was one of three pathogens officially classified by the World Health Organization (WHO) as a Public Health Emergency of International Concern (PHEIC) in the past decade [1]. Currently, the WHO continues to classify it as a “priority pathogen” with epidemic and PHEIC potential [2]. The risk of its resurgence remains high and could be exacerbated by socioeconomic and climatic changes [3]. ZIKV is a unique member of the *Orthoflavivirus* genus with neurotropic and teratogenic potential. It is capable of crossing critical physiological barriers—particularly the blood–brain barrier (BBB) [4], which is primarily formed by brain microvascular endothelial cells (BMECs)—and establishing persistent infection in human BMECs [5]. ZIKV infection can result in severe fetal loss [6], congenital Zika syndrome [7], and neurological syndromes in adults [8]. These severe clinical manifestations are largely attributable to its profound impact on the central nervous system (CNS). ZIKV efficiently targets neural progenitor cells, wherein its capsid protein inhibits Dicer activity and disrupts microRNA biogenesis, thereby impairing corticogenesis [9]. Furthermore, the virus can infect multiple neural cell types, including astrocytes and microglia, triggering sustained neuroinflammation and autophagy dysfunction that underlie long-term cognitive and behavioral deficits [10,11,12]. Critically, BMECs constitute a key portal for viral CNS invasion, and their infection directly contributes to pathogenesis. ZIKV induces the ubiquitin-mediated degradation of Mfsd2a—a critical ω-3 fatty acid transporter in BMECs—thereby compromising BBB integrity and cerebral lipid homeostasis [13]. This endothelial dysfunction facilitates viral dissemination into the brain parenchyma and synergizes with direct virus-mediated neurotoxicity, collectively exacerbating overall neuropathology. To date, no preventive vaccines or specific antiviral therapies for ZIKV are available, and the molecular mechanisms underlying its ability to cross physiological barriers and invade neural tissue remain incompletely understood. Despite the complexity of ZIKV–host interactions, identifying additional host factors involved in ZIKV infection may help address these challenges. Elucidating these host factors could provide foundational insights and facilitate the development of effective preventive and therapeutic strategies.

Viruses such as ZIKV, maintain prolonged interactions with their hosts and have evolved sophisticated strategies to counteract host antiviral immune responses, thereby enhancing their infectivity. The host cellular components involved in this interplay—particularly proteins that either facilitate the viral life cycle or mediate immune defense—are collectively defined as host factors, encompassing both proviral and antiviral factors [14]. Given the limited availability of effective antiviral therapies and the historical focus on viral proteins [15], forward-looking preparedness and response strategies for potential pandemic pathogens—such as ZIKV—should prioritize the development of therapeutic agents targeting host factors essential to the viral infection cycle. Host-targeted antiviral drugs (HTAs) offer a higher barrier to drug resistance and broad-spectrum efficacy [16,17]. Additionally, host factors conserved across multiple viruses are promising targets for universal therapies and broad-spectrum protective vaccines [18]. Driven by practical needs, researchers are increasingly using CRISPR screening technology to systematically perturb genes genome-wide in viral infection models. This approach enables the unbiased identification of critical host factors for various viruses—such as flaviviruses and SARS-CoV-2 [19,20,21]. Indeed, genome-wide CRISPR/Cas9 screening is simple, efficient, and reliable, making it a widely adopted tool in this field. Notably, although the same core technology has been widely used, these studies often show limited overlap, primarily attributed to variations in screening strategies and cellular models. These discrepancies underscore the value of employing diverse CRISPR screening strategies to uncover previously unrecognized host factors closely associated with ZIKV infection.

Animal models that faithfully recapitulate human diseases are indispensable tools for investigating viral infections and underlying pathogenic mechanisms; they also provide critical platforms for developing antiviral therapies and vaccines [22]. The tree shrew (*Tupaia belangeri*) serves as a promising experimental model due to its close evolutionary relationship with primates and has increasingly been recognized as valuable for investigating human viral infections [23]. The ZIKV infection model established in tree shrews effectively recapitulates key clinical and pathological features of human ZIKV infection, such as rash development, viral entry into the brain and testes, and vaginal transmission [24,25,26]. Notably, the tree shrew possesses a fully intact immune system, making it an ideal model for investigating ZIKV interactions with mammalian hosts. However, despite its potential, this valuable model remains underutilized in ZIKV research.

In this study, we established the first genome-wide CRISPR/Cas9 knockout screening platform in the tree shrew model and systematically identified host factors associated with ZIKV infection in BMECs. Furthermore, we characterized the newly identified host factor RNF6, elucidating its specific proviral role and potential mechanisms during ZIKV infection.

## 2. Materials and Methods

### 2.1. Viruses and Cells

The ZIKV strain GZ01 (GenBank accession number KU820898) was initially isolated from a Chinese male patient returning from Venezuela [27] and was provided by Prof. Xueshan Xia for this study. Virus stocks were propagated in Vero cells and titrated using the TCID_50_ assay. The GZ01 strain was inoculated into Vero cells, and significant cytopathic effects (CPE) were observed at least three days post-infection. Both the cells and the supernatants were subsequently harvested. Cell cultures and supernatants were then collected simultaneously. The virus was concentrated using freeze–thaw cycles, centrifugation, and 0.22 µm filtration, clarified by PCR, titrated, and stored at −80 °C. All ZIKV infection experiments were conducted in biosafety level 2 (BSL-2) facilities.

Vero and HEK-293T cells were cultured in DMEM supplemented with 10% fetal bovine serum (FBS; Cell-Box, Changsha, China, CF-01P-02) and 1% penicillin–streptomycin. Tree shrew brain microvascular endothelial cells (BMECs)—a non-genetically modified primary cell line previously established in our laboratory—were cultured in DMEM/F-12 medium supplemented with the same components. Detailed procedures for the isolation, purification, and characterization of tree shrew BMECs are described in the authorized patent (CN115786237B). All cells were maintained at 37 °C in a humidified atmosphere containing 5% CO_2_.

### 2.2. Generation of the Tree Shrew GeCKO Library

To establish a tree shrew genome-wide CRISPR/Cas9 knockout (GeCKO) sgRNA library, we designed sgRNAs targeting all 15,471 protein-coding genes within the tree shrew genome retrieved from the Ensembl database (Taxonomy ID: 37347; https://www.ensembl.org/index.html, accessed on 19 May 2023) at a coverage of six sgRNAs per gene, following established targeting guidelines [28]. Subsequently, a total of 91,297 sgRNAs (including 1000 non-targeting control sgRNAs) were amplified and cloned into the Cas9-lentiGuide-Puro vector to create an all-in-one plasmid library co-expressing Cas9 and sgRNAs. The plasmid library was synthesized by Genscript (Nanjing, China), and the library plasmids were validated by enzymatic digestion and next-generation sequencing.

To establish a tree shrew GeCKO cell library, library plasmids were packaged into lentivirus in HEK-293T cells and transduced into tree shrew BMECs at a multiplicity of infection (MOI) of 0.3 to ensure single sgRNA integration per cell. Puromycin selection (6 μg/mL; GenePharma, Shanghai, China, G04013) was initiated 72 h post-transduction and maintained for 14 days, with fresh medium replenished every 3 days to fully eliminate non-transduced cells. The surviving proliferating cells were designated as the P0 generation. Cas9 expression was confirmed by Western blotting, and sgRNA coverage and homogeneity were assessed by next-generation sequencing. The final BMEC-GeCKO library contains approximately 3 × 10^7^ cells, providing an average coverage of ~300 cells per sgRNA, which ensures adequate representation for the subsequent screen.

### 2.3. Screening of Host Factors Associated with ZIKV Infection Using the Tree Shrew GeCKO Library

The GeCKO screening included three groups: mock-infected library cells (Pc), ZIKV-infected library cells (Pn), and ZIKV-infected wild-type BMECs. Wild-type BMECs served as a positive control for CPE to determine the optimal harvest time. Both Pn cells and wild-type BMECs were infected with ZIKV at an MOI of 5. After 1.5 h, the inoculum was removed, and cells were cultured in DMEM/F-12 medium containing 2% FBS and 1% penicillin–streptomycin at 37 °C and 5% CO_2_. CPE was assessed daily, and Pc served as the negative control. From day 3 post-infection, corresponding to the onset of CPE, medium was refreshed daily to remove dead cells and maintain surviving cell growth. Surviving cells from the Pn and Pc groups were collected once the positive control showed complete CPE with no viable cells remaining. Genomic DNA was extracted, sgRNA fragments amplified, and next-generation sequencing performed on an Illumina HiSeq 3000 (Illumina, Inc., San Diego, CA, USA) to compare sgRNA enrichment between the two groups. The sequencing data were analyzed using the MAGeCK algorithm [29], which ranks candidate genes by robust ranked aggregation (RRA) to identify statistically significant hits. Candidate genes were further functionally annotated using Gene Ontology (GO) and Kyoto Encyclopedia of Genes and Genomes (KEGG) pathway enrichment analyses were performed.

### 2.4. siRNA-Mediated Knockdown of Candidate Genes in BMECs

To ensure effective gene knockdown, small interfering RNAs (siRNAs) targeting candidate genes were designed using homologous transcript sequences from at least two databases: Ensembl (https://www.ensembl.org/index.html, accessed on 20 April 2024), NCBI (www.ncbi.nlm.nih.gov, accessed on 20 April 2024), and Tree Shrew Database (www.treeshrewdb.org, accessed on 20 April 2024). All siRNAs were designed and synthesized by GenePharma (Shanghai, China) and are listed in Table S2. Specific siRNAs or negative control siRNA (si-NC) were transfected into BMECs using a transfection reagent (Engreen, Beijing, China, 4000-3) to knock down endogenous candidate genes.

### 2.5. Generation of a Stable RNF6 Overexpression Cell Line

The coding sequence of tree shrew RNF6 (Transcript: TSDBTID.4671.1; www.treeshrewdb.org, accessed on 24 June 2024) was cloned into the LV5 (EF-1α/GFP&Puro) vector to generate an overexpression plasmid and packaged as lentivirus. The lentivirus was obtained from GenePharma (Shanghai, China) and stored at −80 °C. To establish a stable RNF6-overexpressing cell line in wild-type BMECs, overexpression or control lentiviruses were used to transduce cells at an MOI of 30, with the viral solution incubated for 24 h before being replaced with fresh medium. At 48 h post-transduction, cells were selected with 6 μg/mL puromycin (GenePharma, Shanghai, China, G04013) over two rounds. Successful RNF6 overexpression was confirmed by Western blotting.

### 2.6. Generation of an RNF6 Knockout Clonal Cell Line

To knockout RNF6 in BMECs, we selected a specific sgRNA from our sgRNA library. This plasmid containing sgRNA (5′-CAAAGCGAGGGGCAGCGATT-3′) targeting the tree shrew *RNF6* gene was synthesized by Genscript (Nanjing, China). A total of 0.4 μg of the RNF6-KO plasmid was transfected into tree shrew BMECs using Engreen transfection reagent (Engreen, Beijing, China, 4000-05). Polyclonal KO cells were subjected to limiting dilution to isolate monoclonal RNF6-KO clones. Among the resulting clones, the one with the highest knockout efficiency, as confirmed by Western blotting, was expanded to establish the stable RNF6-KO cells.

### 2.7. CCK-8 Assay

A CCK-8 assay was used to evaluate the impact of RNF6 deletion on cell proliferation. The viability of parental wild-type BMECs (WT) and RNF6-KO cells was compared at 24, 48, and 72 h post-seeding. Cells were seeded in 96-well plates at 2000 cells/well, incubated at 37 °C, and treated with 10 μL of CCK-8 solution per well before measuring absorbance at 450 nm. WT cells served as the control, RNF6-KO cells as the experimental group, and wells with only CCK-8 reagent and culture medium as the blank. Relative cell viability was calculated as: (ODexperiment − ODblank)/(ODcontrol − ODblank) × 100%, with data normalized to the control group.

### 2.8. Generation of a Stable RNF6-Rescue Cell Line

To rescue RNF6 expression in the previously established RNF6-KO cell line, cells were transduced with lentiviral particles carrying the RNF6 coding sequence at an MOI of 20. After 48 h post-infection, cells were selected with 6 μg/mL puromycin and passaged to establish a stable RNF6-rescue (RNF6-RE) cell line.

### 2.9. ZIKV Infection

In this study, ZIKV GZ01 was used to infect various cell types. Viral inoculation and sample collection were standardized: cells were inoculated with the virus at a specific MOI in a maintenance medium containing 2% FBS and incubated for 1.5 h. The inoculum was then removed, and cells were washed three times with PBS to remove unbound virus. Fresh DMEM/F-12 medium with 2% FBS was added, and cultures were continued. At designated time points, supernatants were collected to measure viral genome copies and titers, and cells were harvested to assess ZIKV protein and host gene expression.

### 2.10. RNA Extraction and Quantitative PCR

Total viral RNA was extracted from supernatants of infected cell cultures at various time points according to the manufacturer’s instructions (QIAGEN, Hilden, Germany, 52906). Real-time PCR for ZIKV genomic RNA was performed with the One-Step Absolute Quantification Kit (Takara, Dalian, China, RR064). Viral genome copies of ZIKV in supernatants were calculated from standard curves generated with known concentrations of ZIKV genomic fragments.

Total cellular RNA was extracted from cell samples according to the manufacturer’s instructions (Seven, Beijing, China, SM132-02). cDNA was synthesized using a reverse transcription kit (Promega, Madison, WI, USA, A2801), and mRNA levels were quantified with TB Green ExTaq reagent (Takara, Dalian, China, RR820). Gene expression was calculated using the 2^−ΔΔCt^ method, with GAPDH as the internal control. All specific primers and probe in this study are listed in Table S2.

### 2.11. Virus Titers

Viral titers in supernatants of ZIKV-infected cell cultures were determined by TCID_50_ assay using Vero cells. Supernatant samples were collected and serially diluted 10-fold in DMEM with 2% FBS. Diluted virus was added to Vero cells in 96-well plates at 100 µL per well. Cells were incubated at 37 °C with 5% CO_2_, and CPE were monitored daily. Viral titers were calculated using Karber’s method and expressed as TCID_50_/mL.

### 2.12. Western Blotting Analysis and Antibodies

Cells were washed three times with PBS and lysed on ice in RIPA lysis buffer (Servicebio, Wuhan, China, G2002). Lysates were centrifuged and boiled at 100 °C for 15 min in 5× SDS-PAGE loading buffer (EpiZyme, Shanghai, China, LT101). Proteins were separated by SDS-PAGE and transferred to PVDF membranes (Millipore, Burlington, MA, USA, IPVH00010). Membranes were blocked with 5% skim milk in TBST (Solarbio, Beijing, China, T1082), incubated with primary antibodies at 4 °C overnight, and then with HRP-conjugated secondary antibodies. Protein signals were detected using a chemiluminescent substrate (Millipore, Burlington, MA, USA, WBKLS0500).

Anti-GAPDH (Servicebio, Wuhan, China, GB15002, 1:1000) and anti-β-Actin (Abcepta, Suzhou, China, AP53385, 1:1000) antibodies were used as internal controls. SpCas9 protein in library cells was detected with anti-SpCas9 antibody (Genscript, Nanjing, China, A01935-40, 1:1000). RNF6 protein was assessed using anti-RNF6 antibody (Proteintech, Wuhan, China, 20437-1-AP, 1:1000). ZIKV nonstructural protein 5 (NS5) was detected with anti-ZIKV NS5 antibody (GeneTex, Irvine, CA, USA, GTX133329, 1:5000). Primary antibodies were visualized using HRP-conjugated anti-mouse IgG (ZenBio, Chengdu, China, 511103, 1:15,000) or anti-rabbit IgG (ZenBio, Chengdu, China, 511203, 1:15,000).

### 2.13. Immunofluorescence Assay

At 48 h post-infection (hpi), ZIKV-infected cells were washed twice with PBS, fixed with 4% paraformaldehyde at room temperature for 30 min, permeabilized with permeabilization solution (Servicebio, Wuhan, China, G1204) for 20 min, and blocked with 3% BSA for 30 min. Cells were then incubated with anti-ZIKV NS5 antibody (GeneTex, Irvine, CA, USA, GTX133329) at 4 °C overnight. After PBS washes the next day, cells were incubated with CY3-conjugated goat anti-rabbit IgG (Servicebio, Wuhan, China, GB21303) at room temperature for 50 min. Nuclei were stained with DAPI (Servicebio, Wuhan, China, G1012) in the dark for 10 min, and slides were mounted with anti-fade mounting medium (Servicebio, Wuhan, China, G1401). Fluorescence images were captured using a fluorescence microscope (Nikon, Tokyo, Japan).

### 2.14. Immunoprecipitation and LC–MS/MS Analysis

To identify RNF6-interacting proteins during ZIKV infection, immunoprecipitation (IP) was performed to enrich binding partners. Wild-type BMECs were infected with ZIKV at an MOI of 0.1 or 1 and harvested at 24, 36, and 48 h post-infection for IP. Uninfected cells served as mock-infected controls. Anti-RNF6 antibody (Proteintech, Wuhan, China, 20437-1-AP) was immobilized on resin-based columns according to the manufacturer’s protocol (Thermo Fisher Scientific, Waltham, MA, USA, 26149). Cell lysates were incubated overnight at 4 °C for co-immunoprecipitation. Eluted complexes were neutralized with 1 M Tris-HCl (pH 9.5) (Beyotime, Shanghai, China, ST790-100 mL) and analyzed by LC-MS/MS (Bruker Daltonics, Billerica, MA, USA) to identify interacting proteins.

### 2.15. Co-IP Assay

Co-immunoprecipitation (Co-IP) assays were performed to validate the RNF6–NS5 interaction. Wild-type BMECs were infected with ZIKV at an MOI of 1 and harvested at 36 hpi. Cells were lysed, and supernatants were collected. Immunoprecipitation was conducted using a resin-coupled centrifugal system according to the manufacturer’s protocol (Thermo Fisher Scientific, Waltham, MA, USA, 26149). Briefly, anti-RNF6 (Proteintech, Wuhan, China, 20437-1-AP) or anti-ZIKV NS5 (GeneTex, Irvine, CA, USA, GTX133329) antibody was immobilized on resin-containing columns. Lysates were incubated with antibody-bound resin overnight at 4 °C for co-immunoprecipitation. The next day, immunoprecipitated complexes were eluted and analyzed by Western blotting with the corresponding antibodies to confirm the interaction.

### 2.16. Confocal Imaging

BMECs were infected with ZIKV at an MOI of 0.1. At 36 hpi, confocal microscopy was used to assess co-localization of RNF6 with mitogen-activated protein kinase 1 (MAPK1). Cells were incubated overnight at 4 °C with primary antibodies against RNF6 (Proteintech, Wuhan, China, 20437-1-AP) or MAPK1 (CST, Danvers, MA, USA, 4695). The next day, cells were incubated with HRP-conjugated goat anti-rabbit IgG (Servicebio, Wuhan, China, GB23303) at room temperature for 50 min, followed by TSA treatment (Servicebio, Wuhan, China, G1231 or G1233). Nuclei were stained with DAPI (Servicebio, Wuhan, China, G1012) in the dark for 10 min. Co-localization was visualized and analyzed using a laser confocal microscope (Nikon Eclipse Ti; Nikon, Tokyo, Japan) and NIS-Elements imaging software (version 4.50).

### 2.17. Proteomic Analysis

To systematically characterize the global impact of RNF6 on the host proteome during ZIKV infection, we performed quantitative proteomic profiling of whole-cell lysates. Wild-type BMECs (WT), RNF6-knockout (KO), and RNF6-overexpressing (OE) cells were infected with ZIKV at an MOI of 0.1. At 36 hpi, cells were harvested, and total proteins were extracted for analysis. The quantitative proteomic analysis was conducted by Genedenovo Biotechnology Co., Ltd. (Guangzhou, China) using LC-MS/MS on a timsTOF Pro2 mass spectrometer (Bruker Daltonics, Billerica, MA, USA).

### 2.18. Phylogenetic Analysis

The RNF6 amino acid sequence for tree shrew was sourced from Tree Shrew Database (www.treeshrewdb.org, accessed on 26 November 2025), and those for human and 12 other representative species were sourced from UniProt (www.uniprot.org, accessed on 26 November 2025). Accession numbers are listed in Table S4. Sequences were aligned in MEGA (version 12.1.1), and the phylogenetic tree was built using Maximum Likelihood with 1000 bootstrap replicates. The tree was visualized in iTOL (version 7.3).

### 2.19. Structural Comparison of the RNF6 Protein

Sequence alignment of RNF6 between tree shrew and human was performed using BLASTp (blast.ncbi.nlm.nih.gov, accessed on 26 November 2025) and Clustal Omega (www.ebi.ac.uk/Tools/msa/clustalo/, accessed on 28 November 2025). The BLASTp-derived alignment score assessed protein conservation. Clustal Omega results were analyzed in ESPript (version 3.2) to annotate conserved residues and visualize secondary structure and sequence conservation. The key structural domain of RNF6 was annotated using InterPro (www.ebi.ac.uk/interpro/, accessed on 28 November 2025). The tertiary structure of RNF6 was predicted with AlphaFold 3 and visualized in PyMOL (version 3.03).

### 2.20. Molecular Docking Simulation

To compare the potential functional roles of the RNF6 protein in tree shrew and human, with a specific focus on the NS5 interaction interface, molecular docking simulations were performed using GRAMM (gramm.compbio.ku.edu, accessed on 30 November 2025) to identify conserved binding sites between RNF6 and ZIKV NS5 in both host species.

### 2.21. Statistical Analysis

Statistical analyses were performed using GraphPad Prism 9.0 software. The quantified data were represented as the mean ± standard deviation (SD). A two-tailed unpaired Student’s *t*-test was used to compare differences between two groups, and one-way ANOVA for multiple-group comparisons. Analyses with *p* < 0.05 were considered significant; significant differences in figures are indicated by asterisks: *, *p* < 0.05; **, *p* < 0.01; ***, *p* < 0.001; ****, *p* < 0.0001.

## 3. Results

### 3.1. Establishment of a Tree Shrew Genome-Wide CRISPR/Cas9 Knockout Library in BMECs

**Figure 1:**
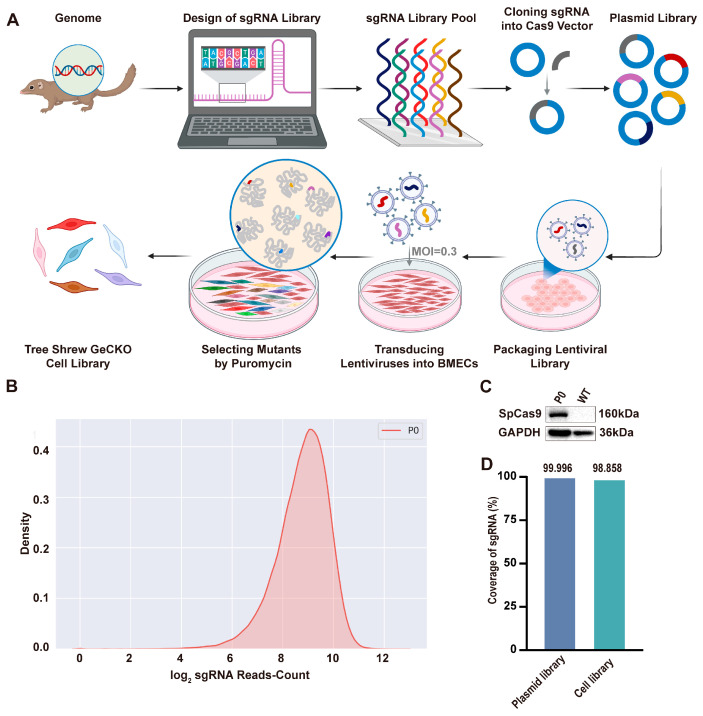
Generation of the tree shrew GeCKO screen cell library. (**A**) Schematic of establishing a tree shrew GeCKO library in BMECs. Created with BioRender.com. (**B**) Evaluation of sgRNA probability distribution in the cell library by next-generation sequencing. (**C**) Assessment of Cas9 expression in BMEC-GeCKO library cells via Western blotting. P0 refers to the first-generation BMEC-GeCKO library cells, and WT refers to the wild-type BMECs. (**D**) Comparative analysis of sgRNA coverage between the tree shrew GeCKO library plasmids and library cells, relative to the designed sgRNA list.

To identify host factors involved in Zika virus (ZIKV) infection, we established a genome-wide CRISPR/Cas9 knockout (GeCKO) library in tree shrew brain microvascular endothelial cells (BMECs) (Figure 1A). Initially, we constructed a whole-genome CRISPR/Cas9 plasmid library for the tree shrew, which contained 91,293 sgRNAs targeting 15,471 coding genes. The library was validated through enzyme digestion (Figure S1A) and next-generation sequencing (Figure S1B), achieving an sgRNA coverage rate of over 99.99% (Figure 1D). The plasmid library was subsequently packaged into lentiviral particles and transduced into tree shrew BMECs. Following antibiotic selection, a stable gene-modified cell population was established, representing the tree shrew BMEC-GeCKO library. Cas9 expression in the library was confirmed by Western blot analysis (Figure 1C). Furthermore, next-generation sequencing of the sgRNA sequences revealed a coverage rate of 98.858% (Figure 1D), and density analysis (Figure 1B) demonstrated a homogeneous distribution of sgRNAs.

### 3.2. Tree Shrew GeCKO Screen Identifies Host Factors Associated with ZIKV Infection

**Figure 2:**
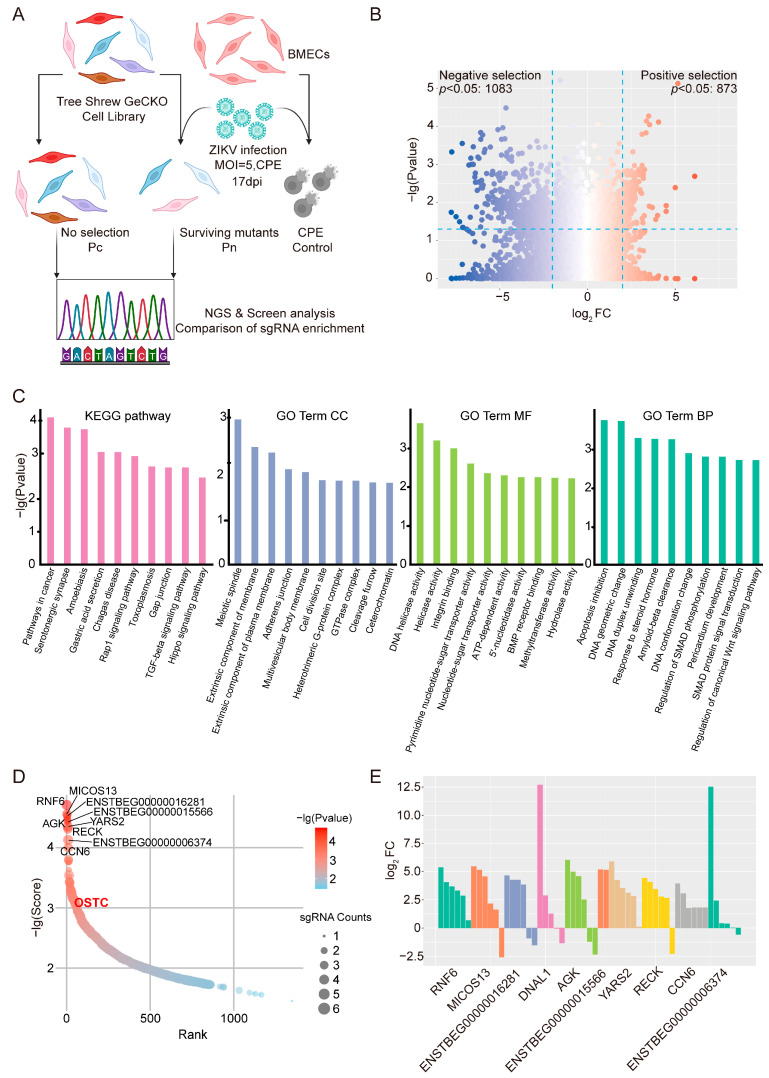
Tree shrew GeCKO screen identifies host factors associated with ZIKV infection. (**A**) Schematic of the tree shrew GeCKO screen for the identification of host factors associated with ZIKV infection. Created with BioRender.com. (**B**) Gene enrichment identified in the GeCKO screen. Red indicates candidate genes through positive selection; blue indicates candidate genes through negative selection. (**C**) KEGG and GO enrichment analyses of candidate genes identified through positive selection in the GeCKO screen. (**D**) Candidate genes through positive selection in the GeCKO screen. The top 10 candidate genes ranked by the MAGeCK-RRA algorithm are labeled in black. The positive control gene is labeled in red. (**E**) Enrichment profiles of individual sgRNAs targeting the top 10 candidate host factors. In CRISPR screening, a lower MAGeCK score indicates a higher gene ranking, with the *x*-axis displaying the top 10 genes ordered by their respective ranks. Note: Uncharacterized tree shrew genes are denoted by their official Ensembl identifiers, beginning with ENSTBEG.

The workflow for the tree shrew GeCKO screen designed to identify host factors associated with ZIKV infection is presented in Figure 2A. The established library cells were infected with the ZIKV GZ01 strain at an MOI of 5. This high screening pressure ensures that all library cells are exposed to ZIKV, minimizing false positives arising from uninfected escapees [30]. Wild-type BMECs infection under the same conditions provide a critical positive control for monitoring cytopathic effects and determining optimal sample timing. Following the isolation of surviving mutant cells (Pn) and mock-infected control cells (Pc), next-generation sequencing was conducted to compare sgRNA enrichment levels between the two populations, enabling the identification of candidate genes. As shown in Figure 2B, further analysis of the sequencing data revealed candidate genes derived from both positive and negative selection. CRISPR screening infers gene function from phenotypes. In the context of our knockout screen, the disruption of proviral host factors for ZIKV infection may confer a survival advantage to mutant cells against ZIKV-induced cell death, enabling their persistence, while loss of antiviral host factors does not. In contrast to negative selection, positive selection is specifically designed to identify genes whose knockout confers a survival advantage [31]. In this study, positive selection provided a more direct approach to identifying proviral host factors for ZIKV. Thus, we focused on the positive selection results to identify host factors essential for ZIKV infection. KEGG and GO enrichment analyses were conducted on the 873 candidate genes identified through positive selection (Figure 2C). These analyses revealed that the identified proviral candidates are broadly enriched in critical biological processes—such as apoptosis inhibition and signal transduction (e.g., SMAD and Wnt signaling)—and in cellular components including membrane-associated structures and adherens junctions. Furthermore, KEGG analysis highlighted the involvement of several key signaling pathways, including Rap1, TGF-β, and Hippo, suggesting that ZIKV extensively exploits these fundamental host survival and signaling networks to establish infection. Figure 2D displays the MAGeCK-RRA scores and ranks of these candidate genes, highlighting the top 10 candidates. Notably, oligosaccharyltransferase complex subunit (OSTC)—a well-established proviral host factor for flaviviruses—emerged as a significant hit in the positive selection (marked in red in Figure 2D). Its successful identification served as a crucial positive control, thereby validating the robustness and reliability of our screening platform. The enrichment patterns of individual sgRNAs targeting these 10 candidates are shown in Figure 2E. Notably, all six sgRNAs targeting RNF6 exhibited significant enrichment (Figure 2E), indicating high on-target efficacy and minimal off-target effects during the screening process. Integrated analysis of fold change and statistical significance (*p*-value) from sgRNA enrichment data (Table S1) further confirmed RNF6 as the top-ranked candidate host factor. The complete results of the positive selection screen are provided in Table S1.

### 3.3. RNF6 Functions as a Proviral Host Factor That Significantly Enhances Viral Replication During ZIKV Infection

**Figure 3:**
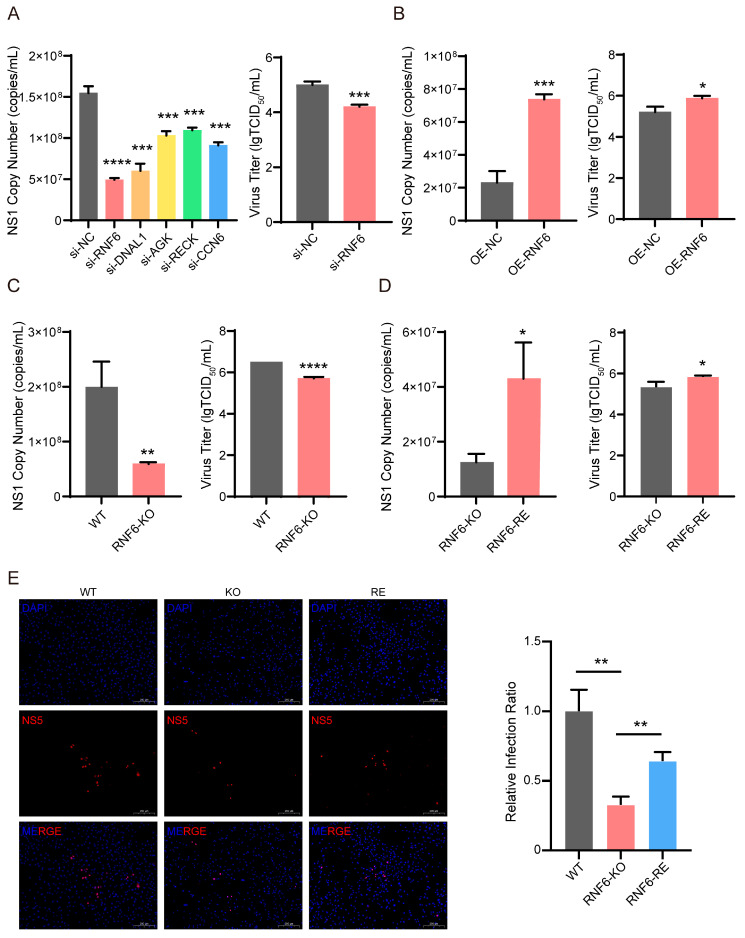
RNF6 functions as a proviral host factor that significantly enhances ZIKV replication. (**A**) Assessment of the effect of siRNA-mediated knockdown of five candidate genes on ZIKV infection. BMECs were transfected with control siRNA (si-NC) or siRNAs targeting RNF6, DNAL1, AGK, RECK, or CCN6 for 48 h, then infected with ZIKV (MOI = 0.01) for 48 h. ZIKV genomic RNA in cell culture supernatants was quantified by NS1 copy number. Viral titers in supernatants were quantified using the TCID50 assay. (**B**) Assessment of the effect of RNF6 overexpression on ZIKV infection. Negative control (OE-NC) and RNF6-overexpressing (OE-RNF6) cells were infected with ZIKV (MOI = 0.01). At 48 hpi, viral genomic RNA and viral titers in supernatants were quantified. (**C**) Assessment of the effect of RNF6 gene knockout on ZIKV infection. Parental wild-type BMECs and RNF6-knockout cells (RNF6-KO) were infected with ZIKV (MOI = 0.1). At 48 hpi, viral genomic RNA and titers in supernatants were quantified. Error bars represent standard deviation of biological replicates; the WT group lacks an error bar as its three replicates had identical measurements (SD = 0). (**D**,**E**) Assessment of the effect of RNF6 restoration on ZIKV infection. RNF6-KO and RNF6-RE cells were infected with ZIKV (MOI = 0.01). At 48 hpi, viral genomic RNA and titers in supernatants were quantified (**D**), and NS5 protein levels in wild-type BMECs, RNF6-KO, and RNF6-RE cells were assessed by immunofluorescence (**E**). Representative fields of view are shown on the left, with the quantification of infection rates on the right. Significant differences are indicated by asterisks: *, *p* < 0.05; **, *p* < 0.01; ***, *p* < 0.001; ****, *p* < 0.0001.

GeCKO screening is a high-throughput, phenotype-based approach widely utilized to identify host factors essential for viral infection. However, technical limitations such as potential off-target effects and insufficient sequencing coverage may yield false positives, necessitating functional validation of candidate genes. To validate the efficacy of this GeCKO screen, particularly the positive selection results, we selected five genes—*RNF6*, *DNAL1*, *AGK*, *RECK*, and *CCN6*—from the top 10 candidates identified by positive selection for preliminary functional validation. Given the current functional annotation limitations of the tree shrew genome, these specific genes were selected because their human homologs have relatively well-defined biological backgrounds. Performing an initial siRNA-based validation on these candidates helps confirm the reliability of the screening platform. Moreover, positive results from these targets—should they be obtained—will provide a solid foundation for future mechanistic studies exploring their roles in ZIKV infection, while the other top candidates identified in the screen also represent a valuable resource for subsequent investigation. Accordingly, gene expression was knocked down using siRNA to investigate whether the identified candidate genes promote ZIKV infection. As shown in Figure 3A, under the same infection conditions (MOI = 0.01, 48 hpi), viral genomic copies were significantly reduced in BMECs transfected with siRNAs against each of the five genes compared to cells treated with a non-targeting control siRNA (si-NC). It is noteworthy that knockdown of RNF6—the top hit from positive selection—using gene-specific small interfering RNA (si-RNF6) resulted in a significant reduction in both viral genomic copies and viral titers (Figure 3A and Figure S2). Obviously, these results indicate that RNF6 serves as a proviral host factor in ZIKV infection.

To further validate the proviral role of RNF6 in ZIKV infection, we infected a stable RNF6-overexpressing cell line (OE-RNF6) established in BMECs (Figure S3) with ZIKV at an MOI of 0.01 and quantified both viral genomic copy number and titer in the cell culture supernatant at 48 h post-infection. Our results demonstrated a significant enhancement in ZIKV production in OE-RNF6 cells (Figure 3B), indicating that RNF6 facilitates the viral life cycle. These findings are consistent with prior loss-of-function data, supporting RNF6 as a critical host factor that promotes ZIKV replication in BMECs.

Considering the limitations of siRNAs in completely suppressing target protein expression, and to comprehensively assess the role of RNF6 in ZIKV infection, we generated a stable RNF6 knockout cell line (RNF6-KO) using CRISPR/Cas9 (Figure S4), with an sgRNA derived from our sgRNA library. Genetic ablation of RNF6 substantially reduced ZIKV infection in BMECs without affecting cell proliferative capacity (Figure S5). After a 48-h infection at an MOI of 0.1, viral genomic copies and titers in RNF6-KO cells were significantly lower than those in wild-type BMECs (WT) (Figure 3C). Furthermore, using the RNF6-KO background, we reintroduced RNF6 to establish the RNF6-rescue cell line (RNF6-RE). The reintroduction of RNF6 restored ZIKV replication in BMECs. Under the same infection conditions, viral copy number, titer, and protein expression levels in RNF6-RE cells were markedly higher than those in RNF6-KO cells (Figure 3D,E). Collectively, these results provide strong evidence that RNF6 functions as a proviral host factor that enhances viral replication during ZIKV infection in BMECs.

### 3.4. RNF6 Interacts with ZIKV NS5 and Negatively Regulates the Type I Interferon and MAPK Signaling Pathways

**Figure 4:**
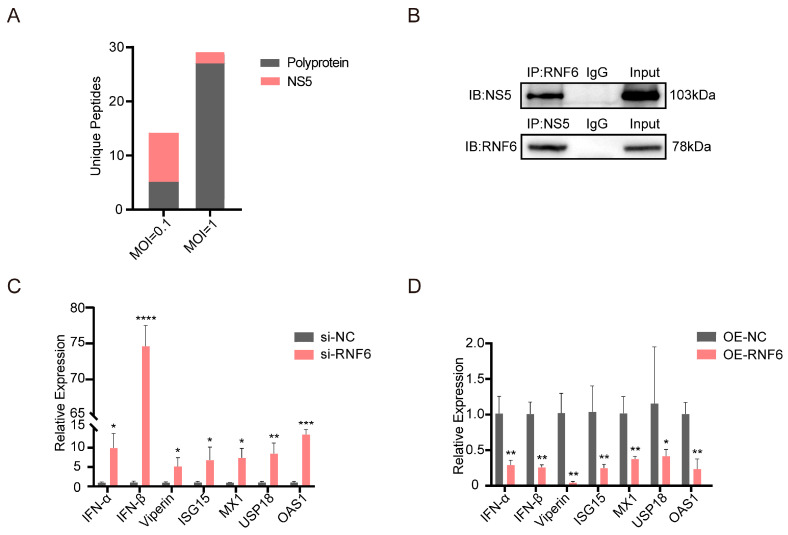
RNF6 interacts with ZIKV NS5 and negatively regulates the type I interferon pathway. (**A**,**B**) RNF6 interacts with ZIKV NS5. (**A**) IP-MS shows RNF6 forms a complex with ZIKV NS5 at 36 hpi (MOI = 0.1 and 1). (**B**) Co-IP confirms the specific interaction between RNF6 and NS5 at 36 hpi. (**C**,**D**) RNF6 is a potential negative regulator of the type I interferon signaling pathway. (**C**) RT-qPCR analysis of IFN-I and ISGs mRNA levels in BMECs following siRNA-mediated knockdown of RNF6. (**D**) RT-qPCR analysis of IFN-I and ISGs mRNA levels in BMECs stably overexpressing RNF6. All cells were infected with ZIKV (MOI = 0.01, 48 hpi). GAPDH was used for normalization. Significant differences are indicated by asterisks: *, *p* < 0.05; **, *p* < 0.01; ***, *p* < 0.001; ****, *p* < 0.0001.

Ring finger protein 6 (RNF6), a RING (Really Interesting New Gene) family E3 ubiquitin ligase, is commonly involved in protein post-translational modification [32,33]. However, its functional role and underlying mechanisms during viral infection remain uncharacterized. To investigate how RNF6 promotes ZIKV replication, we employed immunoprecipitation (IP) to identify RNF6-interacting proteins during ZIKV infection. We performed a combined immunoprecipitation-mass spectrometry (IP-MS) to analyze ZIKV-infected BMECs at MOIs of 0.1 and 1 and time points of 24, 36, and 48 h post-infection. IP-MS results revealed that during ZIKV infection, RNF6 interacts with both host and viral proteins, forming complexes (Table S3). Notably, IP-MS detected the ZIKV NS5 protein in ZIKV-infected BMECs at both MOI 0.1 and 1 at 36 hpi (Figure 4A, Table S3). NS5 is a well-characterized non-structural protein essential for ZIKV immune evasion and replication [34]. To validate the interaction between RNF6 and NS5, co-immunoprecipitation (Co-IP) assays were performed. As shown in Figure 4B, immunoblotting confirmed a specific interaction between RNF6 and NS5 in ZIKV-infected BMECs. The type I interferon (IFN-I) signaling pathway is central to innate immunity against ZIKV infection. However, ZIKV NS5 disrupts multiple key steps in this pathway, thereby suppressing IFN-I production and downstream antiviral immune responses [35]. Consequently, we assessed the regulatory role of RNF6 in modulating the expression of IFN-I and downstream interferon-stimulated genes (ISGs) during ZIKV infection. Specific siRNA-mediated knockdown of RNF6 significantly increased the mRNA levels of IFN-I and ISGs in BMECs (Figure 4C), while its overexpression reduced them (Figure 4D). Together, these results support the hypothesis that RNF6 interacts with ZIKV NS5 and negatively regulates the IFN-I pathway during ZIKV infection, thereby facilitating viral replication in BMECs.

**Figure 5:**
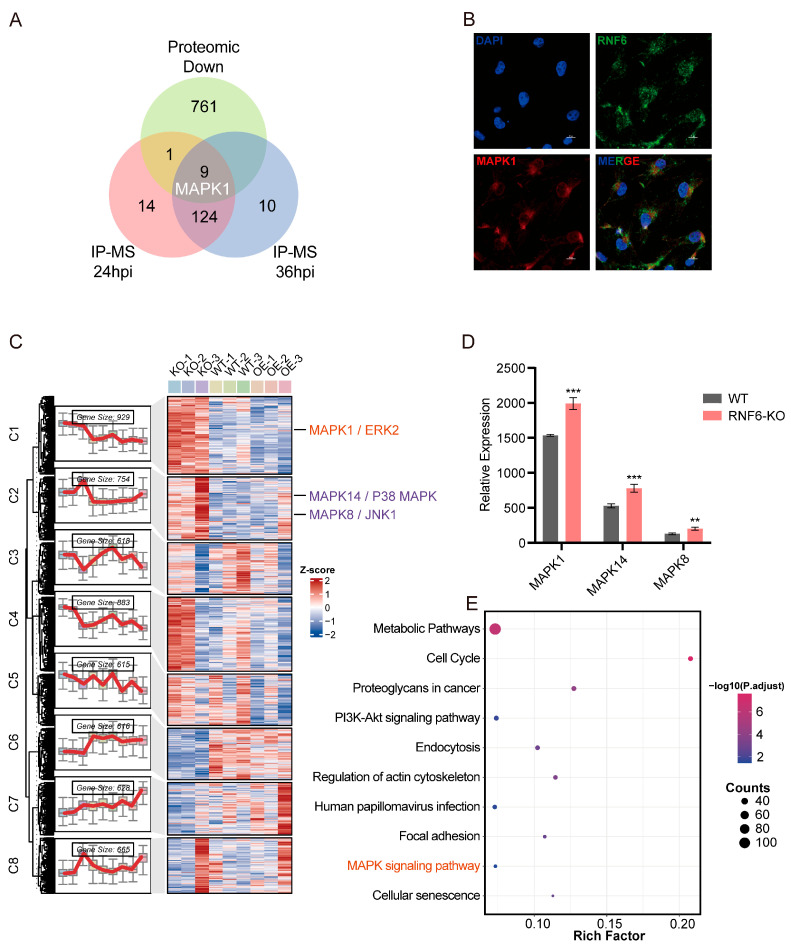
RNF6 also interacts with host MAPK1 and regulates the MAPK signaling pathway. (**A**) Venn diagram analysis integrating IP-MS and proteomic data. IP-MS demonstrates that RNF6 interacts with MAPK1 at both 24 hpi and 36 hpi. Proteomic analysis identifies MAPK1 as a downregulated protein in the WT group compared to the KO group at 36 hpi, revealing an overlap between the two datasets. (**B**) Confocal imaging was employed to assess the subcellular localization of RNF6 and MAPK1 in ZIKV-infected BMECs at 36 hpi. (**C**–**E**) RNF6-knockout (KO), wild-type (WT), and RNF6-overexpressing (OE) BMECs were infected with ZIKV under the same conditions (MOI = 0.1, 36 hpi, *n* = 3) and collected for proteomic analysis. (**C**) Clustering heatmap of all expressed proteins across the three experimental groups. (**D**) Quantitative comparison of MAPK1, MAPK14, and MAPK8 protein levels between the KO and WT groups. Significant differences are indicated by asterisks: **, *p* < 0.01; ***, *p* < 0.001. (**E**) Top 10 enriched pathways from differentially expressed proteins in the KO and WT groups.

Furthermore, IP-MS results revealed that during ZIKV infection, RNF6 interacts with multiple host proteins, including MAPK1, a key member of the mitogen-activated protein kinase (MAPK) family [36]. As illustrated in the Venn diagram (Figure 5A, Table S3), MAPK1 interacted with RNF6 in ZIKV-infected BMECs at both 24 hpi and 36 hpi. Immunofluorescence confocal imaging further confirmed the co-localization of RNF6 and MAPK1 in BMECs during ZIKV infection (MOI = 0.1, 36 hpi) (Figure 5B). To comprehensively characterize RNF6-mediated alterations in the host proteome during ZIKV infection, we performed a global proteomic analysis of whole-cell lysates derived from RNF6-knockout, wild-type, and RNF6-overexpressing BMECs infected under the same conditions (ZIKV MOI = 0.1, 36 hpi). Figure 5C illustrates the RNF6-mediated alterations in the protein expression profile of ZIKV-infected BMECs mediated by RNF6. Notably, the abundance of the host protein MAPK1, previously identified by IP-MS, was found to be negatively correlated with RNF6 expression (Figure 5C). The integration of datasets derived from IP-MS and proteomic analysis converged on MAPK1 (Figure 5A), underscoring its functional significance. These findings collectively indicate that RNF6 directly interacts with MAPK1 at the protein level and suppresses its expression during ZIKV infection in BMECs. In addition to MAPK1 (ERK2), two other representative members of the MAPK family—MAPK14 (p38 MAPK) and MAPK8 (JNK1)—were identified as differentially expressed proteins whose abundance was downregulated by RNF6 (Figure 5C,D). Furthermore, KEGG analysis identified the MAPK signaling pathway as one of the top 10 enriched pathways among proteomics-derived differentially expressed proteins (Figure 5E). Therefore, during ZIKV infection of BMECs, RNF6 suppresses multiple key components of the MAPK signaling pathway at the protein level, thereby modulating this cascade.

### 3.5. Evolutionary and Structural Analyses Support a Conserved Proviral Role for Human RNF6 in ZIKV Infection

**Figure 6:**
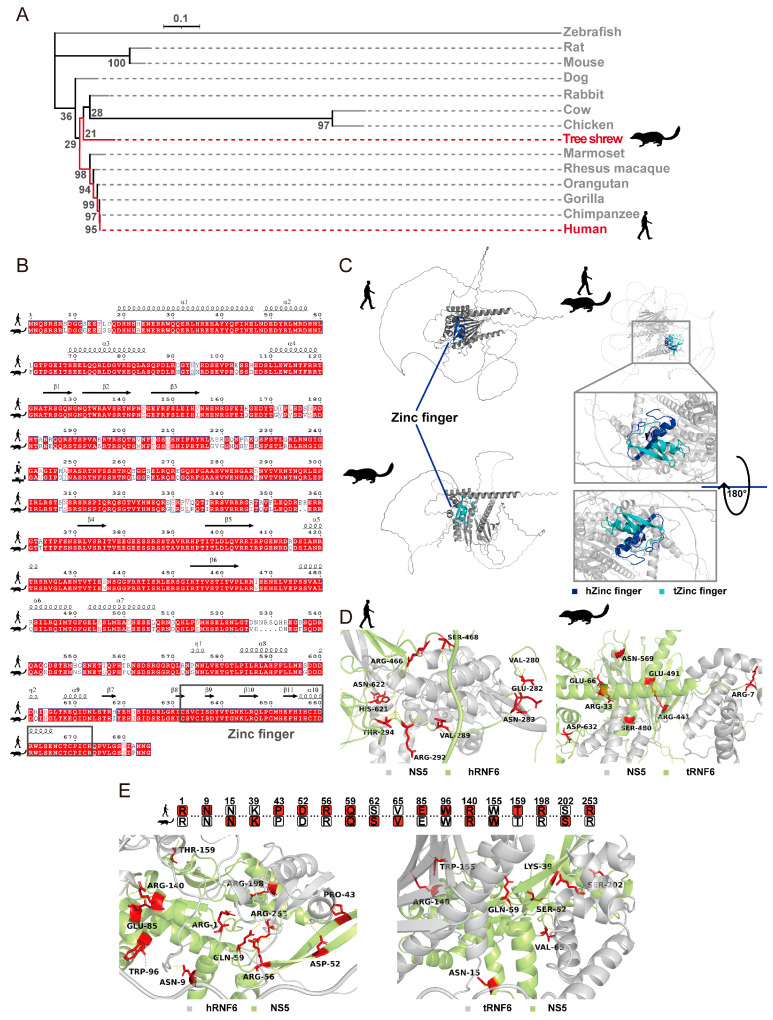
Comparative evolutionary and structural analyses of the RNF6 protein in tree shrew and human. (**A**) A phylogenetic tree of RNF6 proteins from multiple species was constructed using the Maximum Likelihood method. The evolutionary relationship between tree shrew and human is highlighted in red. (**B**) Conservation of the RNF6 protein between tree shrew and human is presented with structural annotations: conserved regions in red; α-helices as wavy lines, β-sheets as arrows. Gray box marks the key structural domain. (**C**) The tertiary structures of RNF6 proteins in the tree shrew and human were predicted using AlphaFold 3, and their structural conformations were directly compared to assess structural conservation of RNF6 between the two species. Their key structural domains were highlighted in blue (human) and cyan (tree shrew), respectively. (**D**,**E**) Molecular docking simulations between tree shrew or human RNF6 and ZIKV NS5 were performed in both orientations. In (**D**), RNF6 is the receptor and NS5 the ligand; in (**E**), NS5 is the receptor and RNF6 the ligand. Potential binding sites are highlighted in red.

To evaluate the translational potential of the host factors identified in our tree shrew model and to lay a theoretical foundation pending future empirical validation in human-derived cells and in vivo models, we first assessed the evolutionary and structural conservation of RNF6. We performed a phylogenetic analysis using the amino acid sequences of RNF6 from tree shrew, human, and 12 other representative species. A maximum likelihood phylogenetic tree was constructed to evaluate the evolutionary relationships of RNF6 across species, with particular focus on the evolutionary proximity between tree shrew and human. As shown in Figure 6A, the tree revealed that the tree shrew, though a non-primate, is closely related to primates, including humans, and shares a relatively recent common ancestor with them. This phylogenetic closeness supports the unique evolutionary position of tree shrew and suggests conservation of the RNF6 protein between human and tree shrew. To specifically evaluate the conservation of the RNF6 protein between the two species, we compared their amino acid sequences and found 87% identity and 92% similarity. Figure 6B aligns conserved regions and secondary structures, revealing high sequence homology, particularly within the Zinc finger domain—the key functional motif of RNF6. This domain recruits E2 enzymes, is essential for E3 ubiquitin ligase activity, and mediates protein–protein interactions. The predicted tertiary structures (Figure 6C) further confirm the structural conservation of RNF6 between the two species; therefore, these collective findings substantiate the conservation of RNF6 in human and tree shrew.

Given that this study identifies tree shrew RNF6 interacting with NS5 during ZIKV infection, we performed bidirectional molecular docking simulations targeting the RNF6–NS5 interaction interface to map binding sites in human and tree shrew orthologs, enabling a direct comparison of their functional potential. These simulations successfully identified potential interaction sites regardless of whether RNF6 (Figure 6D) or NS5 (Figure 6E) was modeled as the receptor. Notably, in Figure 6E, where RNF6 was treated as the ligand and NS5 as the receptor, human and tree shrew RNF6 both targeted two conserved binding residues on NS5, Gln-59 and Arg-140. This interface conservation strongly supports functional similarity between the two orthologs and suggests a conserved role for human RNF6 in ZIKV infection. Overall, our results demonstrate evolutionary conservation and structural similarity of RNF6 between human and tree shrew. Combined with its proviral function in tree shrew cells, these comparative and predictive findings strongly support human RNF6 as a conserved proviral host factor during ZIKV infection, thereby rationalizing our ongoing independent efforts to systematically characterize its function in human systems.

## 4. Discussion

The interaction between Zika virus and host factors is highly complex, and the diversity and mechanisms of these factors may extend beyond our current understanding. Tree shrew, despite being less standardized than mice, is a more suitable small animal model than type I interferon-deficient mice for studying ZIKV–host interactions due to its closer phylogenetic relationship to human and intact immune system. CRISPR/Cas9 knockout screening is a proven approach for identifying critical host factors in viral infections [21]. It requires less comprehensive genome annotation than CRISPR activation (CRISPRa) and produces stronger phenotypes than CRISPR interference (CRISPRi). Therefore, we established a genome-wide CRISPR/Cas9 knockout (GeCKO) library as the first CRISPR screening resource for tree shrew species. To minimize off-target effects in screening, the tree shrew sgRNA library targets each gene with six independent sgRNAs, matching the redundancy of the commercial human GeCKO library (GeCKO v2) [28]. The GeCKO library was built in BMECs, which are critical target cells for ZIKV infection and central to its neuropathogenesis. ZIKV crosses the BBB to infect the central nervous system (CNS)—a pivotal step in neuroinvasion [37,38]. While the underlying mechanisms remain incompletely understood, accumulating evidence supports productive ZIKV infection and replication in BMECs [39,40]. Previous ZIKV CRISPR screens did not use BBB-derived cells, resulting in a lack of identification of host factors involved in the infection of this barrier. Therefore, this GeCKO screen identified RNF6 and other candidate host factors, offering new insights into ZIKV infection and pathogenesis in BMECs.

GeCKO screening is unbiased and high-throughput, enabling detection of both known and novel host factors. It favors identifying dependent host factors essential for viral infection, as cells with inactivating mutations can resist infection and survive under selective pressure. However, it is important to recognize that this survival-based screening approach inherently limits the detection of genes essential for cell viability, because loss of such genes causes cell death or growth arrest—and thus depletion—during pre-infection library expansion. Nevertheless, the utility of this strategy is well-established. For instance, in a GeCKO screen for HIV, Park et al. not only validated known dependent host factors such as CD4 and CCR5, but also identified three novel host factors—TPST2, SLC35B2, and ALCAM [41]. Similarly, multiple GeCKO screens for SARS-CoV-2 have identified known host factors, including ACE2 and TMPRSS2, while also uncovering numerous novel candidates, such as TMEM41B and TMEM106B [42,43]. While direct gene-level overlap across different CRISPR screens can be limited due to varying cell lines and screening parameters, our tree shrew GeCKO screen successfully identified OSTC. OSTC is a previously recognized proviral host factor that has been frequently enriched in previous genome-wide CRISPR screens for flaviviruses [19]. The capture of this consensus factor not only confirms the reliability of our screening methodology but also supports the physiological relevance of our tree shrew model for discovering novel virus–host interactions. Notably, the top ten candidate genes have not been previously associated with ZIKV, and the majority remain unlinked to any viral infection to date. Variations in screening strategies, cell lines, viral strains, and experimental conditions may contribute to the observed heterogeneity in screening outcomes. Such variability has been documented and widely discussed in previous CRISPR screens. Importantly, this heterogeneity is not a limitation as independent screens help uncover novel host factors without requiring identical experimental designs. Even among published CRISPR screens on flaviviruses using human cells, only limited overlap in hit genes has been reported [19,20]. Collectively, independent CRISPR screens expand our understanding of host factors in ZIKV infection, as demonstrated by the findings of this study. To gain deeper insights into the biological significance of host factors, meta-analysis of these independent CRISPR screens could represent a rational next step [20].

RNF6 is a RING family E3 ubiquitin ligase. Most studies to date indicate that RNF6 promotes the progression of various malignant tumors and is associated with chemotherapy resistance [32,44,45]. RNF6’s role in flavivirus infection has not previously been reported. Notably, transcriptomic analyses from public databases (such as the Human Protein Atlas and the Tree Shrew Database) reveal that RNF6 is highly expressed in the brain and testis. This tissue-specific enrichment perfectly mirrors the well-documented neurotropism and reproductive tract persistence of ZIKV [10,46], providing a strong physiological basis for its involvement in ZIKV pathogenesis. Our study extends the current understanding of RNF6’s biological functions. In this tree shrew GeCKO screening, RNF6 was the top hit under positive selection, and its proviral role was confirmed by comprehensive functional assays. These results not only highlight the utility of positive selection in identifying dependent host factors but also confirm the effectiveness of our GeCKO screening. Importantly, such CRISPR screens inherently identify factors essential for overall ZIKV infection. While our validation of RNF6 confirmed its specific role during viral replication, the exact roles of other candidate genes could span any stage of the viral life cycle (including entry, translation, replication, assembly, and release). Future studies are required to explore these additional hits to dissect their precise life-cycle targets.

This study identifies RNF6 as a novel critical host factor in viral infection. Indeed, several E3 ubiquitin ligases have previously been recognized to modulate innate antiviral immune responses through ubiquitination. Importantly, these E3s have dual roles: certain E3s, such as TRIM25 [47], TRIM38 [48], TRIM21, and TRIM14 [49], enhance antiviral defense by positively regulating innate immune signaling, while others, like RNF5 [50], RNF144B [51], and MARCH5 [52] exert negative regulatory effects to prevent excessive immune activation. Viruses frequently hijack these negative regulators to subvert host defenses, representing a well-established immune evasion strategy [53]. The specific mechanism of RNF6 in ZIKV infection may be complex; however, RNF6-mediated regulation of ZIKV replication in BMECs may involve modulation of the antiviral innate immune response. The type I interferon signaling pathway constitutes a central part of innate immunity and drives adaptive immune activation [54]. It is essential for host defense against ZIKV and other *Orthoflavivirus* infections [55]. Conversely, nearly all members of *Orthoflavivirus* have evolved diverse mechanisms to evade this pathway [35]. Their non-structural proteins target multiple stages of the type I interferon pathway, with NS5 acting as the most potent antagonist [56]. NS5, the largest and most conserved protein of ZIKV, performs dual functions in viral RNA replication and immune evasion [57]. ZIKV NS5 interferes with multiple steps of the type I interferon pathway, including pathogen sensing, interferon induction, and JAK/STAT signal transduction [35,58]. Its nuclear localization is critical for inhibiting IRF3 activation and subsequent type I interferon production [59]. Overall, ZIKV NS5 effectively antagonizes type I interferon immunity, facilitating viral infection and replication. A recent study has demonstrated that type I interferon signaling in BMECs—the central structural and immunological components of the BBB—is crucial for BBB integrity and resistance to ZIKV infection. However, ZIKV-encoded proteins weaken this protection by blocking this pathway [60].

In this study, we identified a direct interaction between RNF6 and ZIKV NS5 in BMECs, as well as the role of RNF6 in negatively regulating the type I interferon pathway. Interestingly, a recent study by Sun et al. reported that RNF6 promotes antiviral responses by enhancing the expression of ISGs in myeloid cells [61]. This apparent discrepancy likely reflects fundamental differences among the experimental systems, including the viral infection models (ZIKV versus VSV), host species (tree shrew versus human), and specific cell types (barrier cells such as BMECs versus specialized immune cells such as myeloid cells). The dual roles of RNF6 in cancer biology are well documented: it exerts opposing effects in different cancer types, predominantly promoting tumorigenesis in most cases, yet exhibiting tumor-suppressive functions in specific contexts [62,63]. These precedents further corroborate that the functions and mechanisms of RNF6 must be interpreted within specific biological contexts, suggesting that RNF6 likely exhibits contextual plasticity during viral infections as well. Given the evolutionary capacity of ZIKV to evade host immunity, the direct interaction between RNF6 and NS5 strongly points to a virus-specific hijacking mechanism, wherein ZIKV exploits RNF6 to antagonize the IFN pathway rather than allowing it to exert an antiviral role. Importantly, despite the opposing viral phenotypes observed, both studies independently validate the link between RNF6 and the type I IFN signaling pathway, underscoring its role as a critical modulator of antiviral immunity—a significant step forward given the currently limited understanding of RNF6 in viral infections. Therefore, while our findings elucidate the host-centric mechanisms by which ZIKV establishes infection in BMECs, we cautiously propose that the modulation of the IFN pathway is one of the potential mechanisms underlying the proviral function of RNF6, which warrants more extensive investigation across diverse viral infections. Further studies, particularly those systematically comparing RNF6 functions across different species, cell types, and viral infections, will be crucial for comprehensively elucidating its context-dependent mechanisms of action.

RNF6 regulates several key signaling pathways such as JAK2/STAT3, TGF-β1/c-Myb, Akt/mTOR, ERα/Bcl-xL, Wnt/β-catenin, and MAPK/ERK through ubiquitination, as demonstrated in studies of multiple malignant tumors [64,65,66,67]. In addition to the type I interferon signaling pathway, this study highlights the involvement of RNF6 in the regulation of the MAPK signaling pathway during ZIKV infection. The MAPK signaling pathway regulates diverse cellular processes through a three-tiered kinase cascade: MAPKKK, MAPKK, and MAPK [68]. In mammalian cells, three major classical MAPK subfamilies have been identified—MAPK (also known as extracellular signal-regulated kinases, ERK), c-Jun N-terminal kinases (JNK), and p38 MAPKs. Each transduces extracellular stimuli, including viral infections, into specific cellular responses [69]. Activation of the MAPK pathway is commonly observed in various viral infections [70]. Flaviviruses also activate MAPK members, especially ERK1/2 and p38 [71,72]. However, the biological functions and molecular mechanisms of this activation remain poorly understood. This study reveals that during ZIKV infection of BMECs, RNF6 mediates host proteomic alterations and regulates key MAPK family members—MAPK1 (ERK2), MAPK14 (p38α), and MAPK8 (JNK1). RNF6 directly interacts with MAPK1 at the protein level and reduces its expression. Moreover, the protein levels of these MAPKs are negatively correlated with RNF6, and differentially expressed proteins are enriched in the MAPK signaling pathway. These findings support that RNF6 interacts with the MAPK1 during ZIKV infection of BMECs and acts as a negative regulator of MAPK1 and the MAPK pathway. Few studies have specifically investigated the MAPK pathway’s role in viral replication; evidence indicates it exerts inhibitory effects on viral infection and replication. Biphasic activation of ERK1/2 and p38 MAPK pathways has antiviral effects against infectious laryngotracheitis virus at specific infection stages [73]. In equine herpesvirus 8 infection, ERK1/2 activation enhances biliverdin’s antiviral activity [74]. In addition, MAPK components can phosphorylate key transcription factors such as NF-κB and STAT1 [70], which regulate antiviral innate immunity, indicating that the pathway may help modulate immune responses during viral infection. The role of the MAPK signaling pathway in ZIKV infection is still unclear. Multiple MAPK family members are activated in ZIKV–targeted cells: in retinal Müller cells, ERK1/2 and p38 MAPK are activated [75]; in human BMECs, p38 MAPK, ERK1/2, and JNK are activated [76]. A multi-omics study also links the MAPK pathway to ZIKV infection [77]. Our findings highlight the role of RNF6 in modulating the MAPK pathway during ZIKV infection. The mechanism by which RNF6 negatively regulates MAPK1 and the MAPK pathway, and how this affects ZIKV replication, remains unclear based on current research and our data. Future investigations could prioritize a systematic characterization of RNF6 and its associated signaling networks, including the MAPK pathway and other immune-relevant pathways such as JAK2/STAT3, which may yield critical insights into the complex mechanisms underlying ZIKV pathogenicity.

The WHO has emphasized the epidemic and PHEIC potential of Zika virus. Nevertheless, no clinically approved specific antiviral therapy is available for ZIKV infection to mitigate this threat. Consequently, urgent efforts are needed to identify additional candidate targets for antiviral development. Drug resistance and limited broad-spectrum activity are major limitations of current direct-acting antivirals (DAAs) [78]. Host-targeting antivirals (HTAs) offer a promising alternative. This GeCKO screening identified RNF6 as a key host factor that interacts with ZIKV NS5 and negatively regulates host antiviral immunity, making it a potential HTA target. Our findings show that RNF6 is conserved between human and tree shrew. Notably, molecular docking simulations predict shared NS5-binding residues—Gln-59 and Arg-140—supporting a conserved proviral role for human RNF6 in ZIKV infection. A recent study developed L4P, an anti-HSV-1 peptide that targets the virus–host interaction interface and restores suppressed host immunity, showing reduced drug resistance and fewer side effects [79]. Since ZIKV NS5 is involved in multiple immune evasion mechanisms, L4P’s design strategy provides a blueprint for the development of anti-ZIKV drugs. The NS5 interaction interface with human RNF6 or other host factors could be a rational target for HTA development. In addition to its potential in developing antivirals, RNF6 may also aid in optimizing ZIKV infection models. Identified in the tree shrew and implicated in various malignant tumors, RNF6 is a candidate whose modulation could facilitate the establishment and refinement of tree shrew–based models for viral infections and tumors.

In summary, we have successfully developed a GeCKO library in the tree shrew model and identified host factors associated with ZIKV infection in BMECs. Our findings reveal RNF6 as a novel and critical host factor that promotes ZIKV replication, potentially through its interaction with ZIKV NS5 and negative regulation of type I interferon and MAPK signaling pathways. Importantly, our data support a conserved proviral role for human RNF6 during ZIKV infection, highlighting the need to further characterize its interaction interface with NS5. This study employs an innovative approach to uncover novel host factors, advances the understanding of ZIKV replication at the BBB, and provides new insights for developing host-directed antiviral therapies.

More broadly, this work introduces a novel paradigm by integrating CRISPR screening with the tree shrew infection model, thereby establishing a powerful platform for dissecting virus–host interactions. We acknowledge that a limitation of the current study is the absence of empirical validation for these host factors in human-derived cells or in vivo models. However, our comparative evolutionary and structural analyses provide a robust theoretical bridge strongly implying that the RNF6-NS5 interaction mechanisms are conserved from tree shrews to humans. Our findings lay a critical foundation and open a new investigative avenue, even as the detailed function of human RNF6 and its specific ubiquitination mechanisms await full validation. The clear subsequent roadmap—which is the focus of our ongoing independent investigations to systematically characterize human RNF6’s function, its interplay with NS5, the associated ubiquitination network, and its therapeutic potential—enables the direct translation of this foundational work. Advancing this platform will be instrumental in unraveling key ZIKV-host regulatory axes, thereby paving the way for targeted antiviral strategies.

## Data Availability

The original contributions presented in this study are included in the article/Supplementary Materials. Further inquiries can be directed to the corresponding authors.

## Supplementary Materials

The following supporting information can be downloaded at: https://www.mdpi.com/article/10.3390/v18030323/s1, Figure S1: Analysis of the GeCKO plasmid library by en-zyme digestion and next-generation sequencing; Figure S2: RT-qPCR analysis of RNF6 mRNA expression in siRNA-treated BMECs; Figure S3: Western blot analysis of RNF6 protein expression in the OE-RNF6 cells; Figure S4: Western blot analysis of RNF6 protein expression in the RNF6-KO cells; Figure S5: The proliferative capacity of RNF6-KO cells was assessed using the CCK-8 assay; Table S1: Genome-wide screen MAGeCK scores based on positive selection; Table S2: List of primers, probe and siRNAs in this study; Table S3: Host and viral proteins interacting with RNF6 during ZIKV infection, identified through IP-MS; Table S4: The RNF6 amino acid sequences used in this study.
