# Supplementary material for: Tree Shrew Genome-Wide CRISPR Screen Identifies RNF6 as a Proviral Host Factor for Zika Virus Replication in Brain Microvascular Endothelial Cells
Source: Viruses. 2026 Mar 5;18(3):323. doi: 10.3390/v18030323 (PMC13030181; doi:10.3390/v18030323)

Supplementary Materials

Supplementary Figures

Figure S1. Analysis of the GeCKO plasmid library by enzyme digestion and next-generation sequencing. (A) Enzyme digestion of the GeCKO library plasmids. Lane M: KB Ladder; Lane 1: library plasmid; Lane 2: library plasmid digested by EcoRl and BamHl. (B)Analysis of sgRNA distribution within the plasmid library using next-generation sequencing.

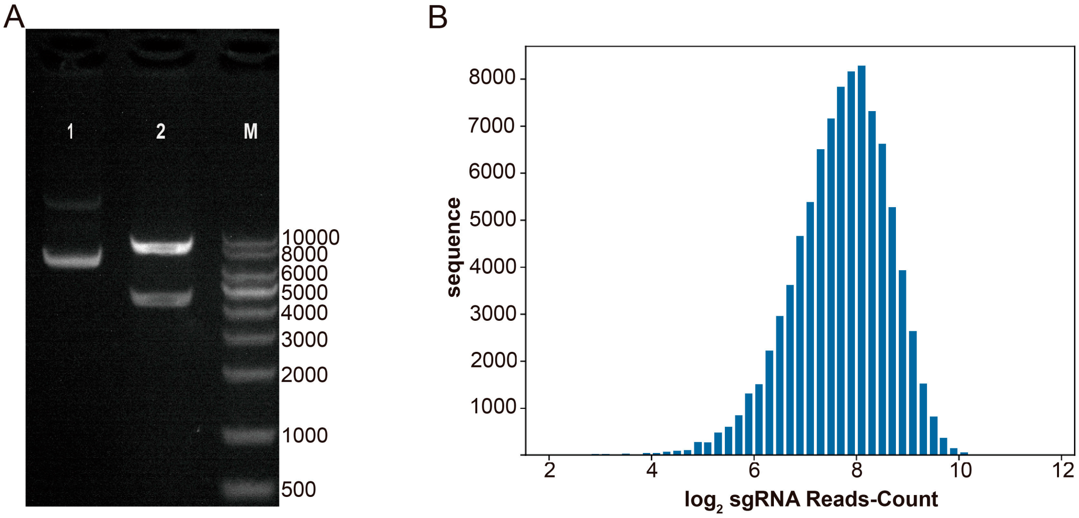

Figure S2. RT-qPCR analysis of RNF6 mRNA expression in siRNA-treated BMECs. Four specific siRNAs targeting the RNF6 gene (RNF6-siRNA 1–4) and a negative control siRNA (NC-siRNA) were respectively transfected into BMECs. RNF6 mRNA expression levels were assessed by RT-qPCR at 24 and 48 hours post-transfection, identifying RNF6-siRNA2 as the most effective. Its sequence, designated si-RNF6, is listed in Table S2.

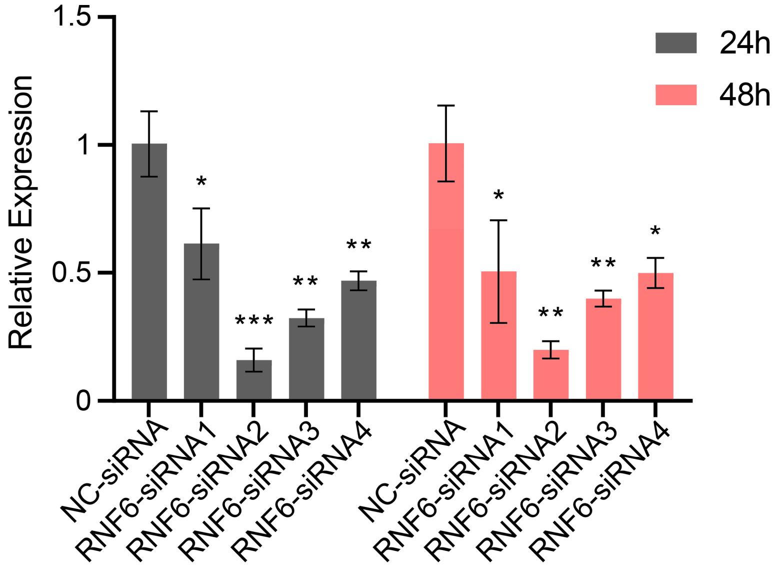

Figure S3. Western blot analysis of RNF6 protein expression in the OE-RNF6 cells. Wild-type BMECs were transduced with lentiviruses to stably overexpress RNF6 (OE-RNF6) or served as negative control (OE-NC). RNF6 overexpression was confirmed by Western blotting. β-actin served as a loading control.

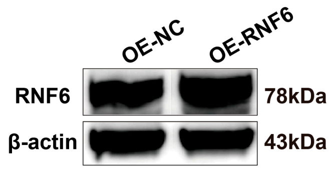

Figure S4. Western blot analysis of RNF6 protein expression in the RNF6-KO cells. Western blot analysis of RNF6 protein expression in parental wild-type (WT) and RNF6-knockout (RNF6-KO) BMECs generated using CRISPR/Cas9. β-actin served as a loading control.

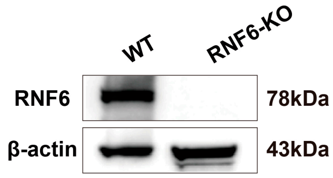

Figure S5. The proliferative capacity of RNF6-KO cells was assessed using the CCK-8 assay. To assess the impact of RNF6 deletion on cell proliferation, the viability of parental wild-type BMECs (WT) and RNF6-KO cells was compared at 24, 48, and 72 hours using the CCK-8 assay.

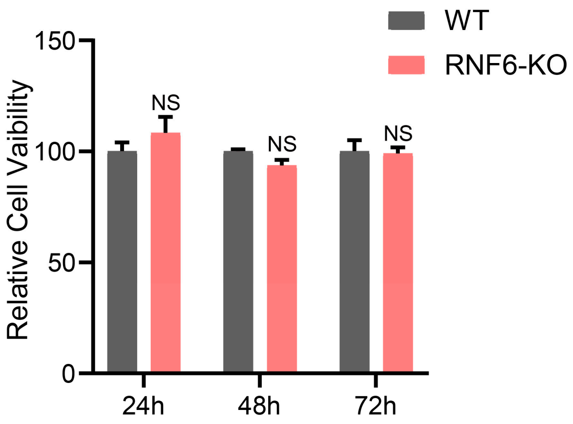

Supplement: Supplementary file 1 [file viruses-18-00323-s001.zip › Supplementary Figures.pdf]
